# Evaluation of resistance modulation in MDR *Pseudomonas aeruginosa* and *Klebsiella pneumoniae* using peppermint oil nanoemulsion: integrating antibacterial assays and molecular modeling

**DOI:** 10.3389/fmicb.2025.1704938

**Published:** 2025-11-26

**Authors:** Sardar Ali, Firasat Hussain, Tehmeena Nousheen, Kashif Rahim, Hamid Majeed, Kamal Niaz, Muhammad Nadeem Khan

**Affiliations:** 1 School of Medical Sciences, Shandong Xiehe University, Jinan, China; 2 Department of Microbiology, Cholistan University of Veterinary and Animal Sciences, Bahawalpur, Pakistan; 3 Department of Food Sciences, Cholistan University of Veterinary and Animal Sciences, Bahawalpur, Pakistan; 4 Department of Pharmacology and Toxicology, Cholistan University of Veterinary and Animal Sciences, Bahawalpur, Pakistan; 5 Department of Cell Biology and Genetics, Shantou University Medical College, Shantou, China

**Keywords:** peppermint oil nanoemulsion, urinary tract infection, *Klebsiella pneumoniae*, *Pseudomonas aeruginosa*, multidrug resistance, nanotechnology, antimicrobial, β-caryophyllene

## Abstract

**Introduction:**

Multidrug-resistant urinary tract pathogens, primarily *Klebsiella pneumoniae* and *Pseudomonas aeruginosa*, represent a significant and growing public health challenge. The overuse of antibiotics has accelerated the development of resistance, creating an urgent need for alternative antimicrobial strategies. This study aimed to evaluate the antibacterial efficacy of a peppermint oil nanoemulsion (PEONE) against clinical multidrug-resistant (MDR) isolates of *K. pneumoniae* and *P. aeruginosa*, with a specific focus on its potential for resistance modulation and its mechanism of action.

**Methods:**

Clinical isolates were obtained from patients with urinary tract infections and their antibiotic susceptibility profiles were determined. The PEONE was prepared using ultrasonic emulsification and characterized, revealing a droplet size of 190.21 ± 0.5 nm and a polydispersity index (PDI) of 0.15 ± 0.021. Antibacterial activity was assessed by determining the minimum inhibitory concentration (MIC) and minimum bactericidal concentration (MBC). Membrane integrity was evaluated using DNA and protein leakage assays, and bacterial killing over time was measured with time-kill kinetics. Furthermore, molecular docking and 100 ns molecular dynamic simulations were performed against β-lactamase enzymes (PDB: 4EXY from *K. pneumoniae*, 6R73 from *P. aeruginosa*) to identify key bioactive components within the nanoemulsion.

**Results:**

The bacterial isolates were resistant to Levofloxacin, Penicillin G, Ceftazidime, and amoxicillin-clavulanic acid (AMC). PEONE demonstrated potent antibacterial activity, with an MIC of 0.1% v/v and an MBC of 0.14% v/v. DNA and protein leakage increased significantly (*p* < 0.05) with higher PEONE concentrations, indicating bacterial membrane disruption. Time-kill assays showed a sustained reduction in bacterial viability over 72 hours, with significant differences emerging after 12 hours of exposure. Molecular docking revealed that caryophyllene, a major component of peppermint oil, had the highest binding affinity against both β-lactamase targets (−9.2 kcal/mol for 4EXY; −7.1 kcal/mol for 6R73). The stability of this binding was confirmed through molecular dynamics simulations.

**Discussion:**

The findings indicate that PEONE is effective at inhibiting and killing MDR *K. pneumoniae* and *P. aeruginosa*. The observed leakage of DNA and proteins suggests that the mechanism of action likely involves disruption of the bacterial membrane, leading to the loss of intracellular contents. This is further supported by the computational data, which identified caryophyllene as a key component with stable binding to resistance-associated β-lactamase enzymes. These results position PEONE as a promising, plant-based alternative for combating antibiotic-resistant urinary tract infections. Further *in vivo* studies are warranted to explore its clinical applicability and safety profile.

## 1 Introduction

Urinary tract infections (UTIs) are among the most common bacterial infections worldwide, affecting over 150 million people annually (Zeng et al., 2022). They represent a significant health burden, particularly among women, with above 50% experiencing at least one episode in their lifetime (Medina and Castillo-Pino, 2019). UTIs can be acquired in both community and nosocomial settings, with *Klebsiella pneumoniae* and *Pseudomonas aeruginosa* emerging as key pathogens, especially in catheterized or immunocompromised patients (Klein and Hultgren, 2020).

Diagnosing and treating UTIs remains challenging due to the variability in clinical presentation ranging from asymptomatic to severe symptomatic cases and the rising prevalence of antibiotic resistance (Frimodt-Møller and Bjerrum, 2023). For instance, resistance to first-line antibiotics like ampicillin and trimethoprim/sulfamethoxazole (SXT) has reached alarming levels, with reports indicating 51 and 29% resistance rates, respectively, in pediatric populations (Adeyemo et al., 2025). This resistance stems from the natural adaptability of bacteria, which evolve defense mechanisms against conventional antibiotics (Adeyemo et al., 2025). Consequently, there is an urgent need for alternative therapeutic strategies that can circumvent resistance while remaining effective against uropathogens.

Nanotechnology has emerged as a promising approach to combat antimicrobial resistance, offering advantages such as enhanced drug stability, bioavailability, and targeted delivery (Bharti and Kumar, 2025). Among nanocarriers, nanoemulsions (NEs) are particularly notable for their small droplet size, high permeability, and ability to improve the solubility and efficacy of antimicrobial agents (Garcia et al., 2022). When combined with bioactive plant-derived compounds, NEs present a sustainable solution to antibiotic resistance, leveraging natural molecules against which bacteria have not yet developed resistance (El-Saadony et al., 2025).

Peppermint essential oil (PEO), rich in menthol, menthone, and other bioactive metabolites, exhibits broad-spectrum antimicrobial, anti-inflammatory, and immunomodulatory properties (Kazemi et al., 2025). The antimicrobial efficacy of PEO derives from its complex composition of bioactive compounds, including menthol, menthone, and caryophyllene, which are thought to act synergistically on multiple cellular targets. Formulating PEO into a PEONE may enhance its therapeutic potential by improving delivery and countering bacterial resistance mechanisms. Despite documented antibacterial effects, PEO’s precise mode of action, especially its capacity to modulate specific molecular pathways conferring resistance, requires further elucidation. The individual contributions of its principal constituents also remain poorly defined.

To address the critical challenge of antibiotic-resistant urinary tract infections, this investigation employs a dual-methodological framework to characterize PEONE as a novel antimicrobial agent. Our objectives are twofold: first, to determine the *in vitro* antibacterial efficacy and resistance-modulating potential of PEONE against multidrug-resistant clinical isolates of *K. pneumoniae* and *P. aeruginosa*; and second, to decode the molecular interactions between key PEONE phytoconstituents and bacterial resistance determinants, specifically beta-lactamase enzymes, through *in silico* docking and dynamic simulation studies, thereby proposing a coherent mechanism of action. Given the escalating threat of antibiotic resistance in UTIs, this study focuses on evaluating peppermint oil nanoemulsions as a novel antimicrobial strategy against resistant strains of *K. pneumoniae* and *P. aeruginosa*. Specifically, the study aims to assess the antibacterial efficacy of PEO nanoemulsions against UTI-causing resistant bacteria and to investigate the potential of PEO nanoemulsions in modulating bacterial antibiotic resistance.

This work is critical in advancing alternative therapies that can alleviate the burden of antimicrobial resistance, ensuring sustainable treatment options for UTIs. By harnessing the synergistic potential of nanotechnology and phytochemicals, this study contributes to the development of innovative, resistance-combating formulations in infectious disease management.

## 2 Materials and methods

### 2.1 Sample collection

The study was conducted at the Postgraduate Laboratory, Department of Microbiology, Faculty of Veterinary Sciences, Cholistan University of Veterinary and Animal Sciences, Bahawalpur. The protocol was approved by the Institutional Ethical Review Committee of Cholistan University of Veterinary and Animal Sciences (Approval No: 1223). Ten mid-stream urine samples were aseptically collected in sterile containers from patients diagnosed with UTIs at Bahawal Victoria Hospital (BVH). Samples were immediately transported to the laboratory under refrigerated conditions (4 °C) and processed within 2 h of collection to minimize bacterial overgrowth. The samples were residual; anonymized specimens obtained after routine diagnostic analysis therefore the requirement for individual patient consent was waived by IERB.

### 2.2 Bacterial isolates and culture conditions

Clinical isolates of *P. aeruginosa* and *K. pneumoniae* were obtained by culturing uncentrifuged urine samples on Cystine Lactose Electrolyte Deficient (CLED) agar (HiMedia, Thane(W)-400604 India). Primary isolates were sub-cultured on Luria Bertani (LB) agar (Oxoid, Hampshire, UK) and maintained at 4 °C for short-term storage. For long-term preservation, isolates were stored in glycerol broth (20% v/v) at −80 °C. The isolates were presumptively identified through Gram staining, catalase-oxidase activity and colony morphology on selective media. A total of twenty clinical isolates, comprising ten *K. pneumoniae* and ten *P. aeruginosa*, were obtained and used in this study.

### 2.3 Antibiotic susceptibility testing

Antimicrobial susceptibility against selective antibiotics Gentamicin (10 μg), Amoxicillin-clavulanic acid (AMC, 30 μg), Ceftazidime (30 μg), Penicillin G (10 units), Imipenem (10 μg), and Levofloxacin (5 μg) purchased from Sigma-Aldrich (MO, United States) was determined through Kirby-Bauer disk diffusion method. MHA plates according inoculated with pathogens were incubated at 37 °C for 24 h, and inhibition zone diameters were measured to determine susceptibility patterns following CLSI 2022 (Gaur et al., 2023).

### 2.4 Preparation of peppermint oil nanoemulsion

PEONE was prepared using an ultrasonic emulsification method adapted from (Majeed et al., 2016) to form an oil-in-water (O/W) nanoemulsion, which facilitates interaction with the aqueous bacterial environment. Briefly, the aqueous phase contained 2% (w/w) Purity Gum Ultra (PGU) a succinylated modified starch (Golden shell, Yuhuan, China) in deionized water, which was hydrated overnight. The oil phase, (peppermint essential oil) purchased from Bioshop Pk (Karachi, Pakistan) with >98% was added to the aqueous phase at 1:4 ratio and homogenized at 13,500 rpm for 2 min forming a preliminary, macro-scale mixture known as a coarse emulsion. The chemical composition is provided in Supplementary Table S1. The coarse emulsion was then processed through an ultrasonic homogenizer (UCP-1200, Hangzhou, China) at 50–150 MPa for 1–20 passes, maintaining temperature at 15 °C using a heat exchanger.

The PEONE was then characterized for droplet size, polydispersity index (PDI), and zeta potential using dynamic light scattering (DLS) (Malvern Zetasizer Nano ZS, UK). The average droplet size was found to be 190.21 ± 0.5 nm with a PDI of 0.15 ± 0.021. The zeta potential was measured to be −25 mV, suggesting good physical stability.

### 2.5 Determination of minimum inhibitory concentration

The minimum inhibitory concentration (MIC) of PEONE was determined by broth microdilution method in 96-well plates. Two-fold serial dilutions (0.04–0.1% v/v) were prepared in MH broth. The nanoemulsion formed a stable, homogenous mixture in the broth without visible phase separation. Each well was inoculated with 10 μL of a fresh bacterial suspension prepared by adjusting the turbidity of an overnight culture of a defined mixture (1:1) of *P. aeruginosa* and *K. pneumoniae* in sterile saline to a 0.5 McFarland standard, followed by dilution in MH broth to achieve a final concentration of approximately 10^4^ CFU/mL and incubated at 37 °C for 24 h. The MIC was defined as the lowest concentration showing no visible growth. Appropriate controls were included in all assays: a growth control (bacterial inoculum in MH broth), a surfactant control (bacterial inoculum in MH broth containing the highest concentration of Purity Gum Ultra used in the nanoemulsion formulations), and a sterility control (uninoculated MH broth). The antibacterial activity of PEONE was assessed relative to these controls.

### 2.6 Minimum bactericidal concentration determination

Minimum bactericidal concentration (MBC) was determined by subculturing 10 μL from clear MIC wells onto MHA plates. After 24 h incubation at 37 °C, MBC was recorded as the lowest concentration showing ≥99.9% killing of the initial inoculum. The MBC/MIC ratio was calculated to determine bactericidal (≤4) or bacteriostatic (>4) activity.

### 2.7 Nucleic acid and protein quantification

To evaluate the membrane disruption potential of PEONE, the leakage of nucleic acids and proteins from treated bacterial cells was quantified by analyzing the cell-free supernatant. Samples for this analysis were taken directly from the wells of the MIC assay (Section 2.5) after the 24-h incubation period. The content from wells showing no visible growth was collected. The samples were centrifuged (10,000 × g, 10 min) to separate the bacterial cells from the supernatant. The resulting cell-free supernatant was used to quantify the leakage of cytoplasmic proteins using the Bradford assay. A standard curve was generated using bovine serum albumin (0.1–1.0 mg/mL). Supernatant samples were diluted 1:100 in PBS, and absorbance was measured at 595 nm in triplicate. Simultaneously, the bacterial pellet was used to evaluate genomic DNA. DNA was extracted from the pellet using the phenol chloroform method (Gautam, 2022) to assess damage associated with membrane disruption. The concentration and purity of the extracted DNA were measured spectrophotometrically using a NanoDrop, with an A260/A280 ratio of 1.8–2.0 indicating pure DNA.

### 2.8 Time-kill kinetics assay

Time-kill studies were performed using the MIC concentration (0.08% v/v) against bacterial suspensions (10^6^ CFU/mL) in MH broth. Viable counts were determined at 0, 3, 6, 9, 12, 24, 48, and 72 h by plating serial dilutions on MHA. Bactericidal activity was defined as ≥3 log_10_ reduction in CFU/mL from initial inoculum.

### 2.9 Molecular docking and dynamic simulations

#### 2.9.1 Target selection and preparation

The crystal structures of two bacterial β-lactamases from *K. pneumoniae* (PDB ID: 4EXY) (King et al., 2012) and *P. aeruginosa* (PDB ID: 6R73) (Softley et al., 2020), were selected as molecular targets. In preparation for docking using AutoDock,^1^ all water molecules, cofactors, and ions were removed from each structure. Polar hydrogen atoms were then added, Gasteiger charges were assigned, and the prepared proteins were exported in the PDBQT file format.

#### 2.9.2 Ligand preparation

A library of 10 primary compounds constituting PEO was selected for *in silico* screening. Their three-dimensional structures were retrieved from the PubChem database and subsequently prepared using AutoDock. This preparation involved energy minimization, the addition of polar hydrogens, and the assignment of rotatable bonds prior to conversion into the PDBQT format.

#### 2.9.3 Docking protocol

Binding affinities and poses of the ligands within each target’s active site were predicted through molecular docking executed in AutoDock Vina (Trott and Olson, 2010). A grid box was parameterized to encapsulate the known active site residues of each protein (4EXY: center_x=, center_y=, center_z=, size_x=, size_y=, size_z=; 6R73: center_x=, center_y=, center_z=, size_x=, size_y=, size_z=) (Forli et al., 2016). The exhaustiveness parameter was set to [e.g., 8 or 16] to ensure conformational sampling robustness. For each ligand, the top nine poses were generated and ranked according to their predicted binding affinity (ΔG, in kcal/mol), where more negative values correspond to stronger binding.

#### 2.9.4 Analysis of docking results

The resulting protein-ligand complexes were visualized within the PyMOL Molecular Graphics System (Schrödinger, LLC) to generate structural representations. A comprehensive analysis of molecular interactions including hydrogen bonding, hydrophobic contacts, and pi-pi stacking was conducted using the BIOVIA Discovery Studio Visualizer (Baroroh et al., 2023).

#### 2.9.5 Molecular dynamics simulations

The stability and dynamic behavior of the top-ranked docking complex(es) were evaluated through molecular dynamics simulations performed with the Desmond software, Schrödinger LLC (Bowers et al., 2006). Initial complex preparation, involving hydrogen bonding network optimization and structural minimization, was conducted using the Maestro Protein Preparation Wizard. An orthorhombic solvation box of TIP3P water molecules was built around the prepared system using the System Builder tool. The system’s charge was neutralized with appropriate counterions, and a physiological ion concentration of 0.15 M NaCl was introduced. Simulations were performed under isothermal-isobaric (NPT) ensemble conditions (1 atm, 300 K) for a duration of 100 nanoseconds, with trajectory frames saved at 100 ps intervals for analysis. The simulation’s stability was quantified by calculating the Root Mean Square Deviation (RMSD) of the protein backbone and the ligand relative to the initial coordinates, utilizing the Simulation Interaction Diagram tool in Desmond (Grant et al., 2021).

### 2.10 Statistical analysis

All experiments were performed in triplicate. Data were analyzed using GraphPad Prism and expressed as mean ± standard deviation. One-way ANOVA was applied to compare the different concentrations of PEONE with the control, followed by Tukey’s *post-hoc* test for multiple comparisons, with a significance level set at *p* < 0.05.

## 3 Results

After 24 h of incubation at 37 °C, *K. pneumoniae* produced large, yellow or yellowish-white colonies. The colonies were highly mucoid and elevated on CLED medium. While *P. aeruginosa* formed medium to large colonies that appeared greenish-blue to bluish-green on MHA medium. Both were gram negative (Supplementary Figure S1).

### 3.1 Antibacterial susceptibility test

The Antibacterial Susceptibility Test (AST) was performed on a polymicrobial mixture of *P. aeruginosa* and *K. pneumoniae* to simulate a challenging, multi-pathogen infection environment. The results showed that the mixture of pathogens was resistant to Levofloxacin, Penicillin G, Ceftazidime, and AMC (Table 1).

**Table 1:** Antibiotic susceptibility profile of *P. aeruginosa* and *K. pneumoniae* mixture.

Antibiotics	Mixture of *P. aeruginosa* and *K. pneumonia*
IMP (10 μg)	22 nm (I)
LEV (5 μg)	0 (R)
P (10 UI)	0 (R)
CN (10 μg)	9 nm (S)
CaZ (30 μg)	0 (R)
AMC (30 μg)	0 (R)

R, Resistant; I, Intermediate; S, Sensitive.

### 3.2 MIC and MBC of nanoemulsion

**Figure 1:**
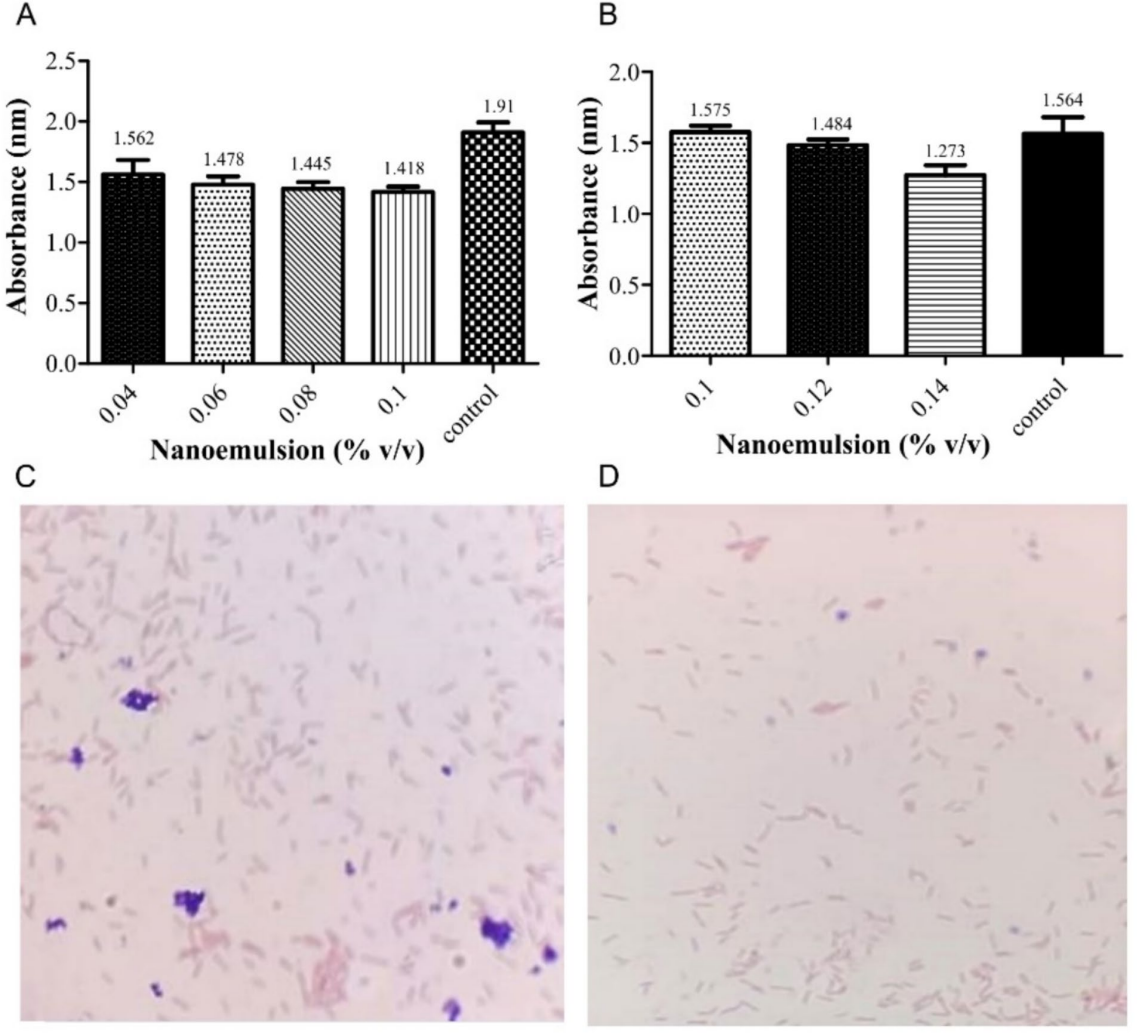
Minimum Inhibitory Concentration (MIC) and Minimum Bactericidal Concentration (MBC) of peppermint oil nanoemulsion against a mixture of *P. aeruginosa* and *K. pneumoniae*. **(A)** Absorbance (nm) measurements of MIC; **(B)** Absorbance (nm) measurements of MBC Microscopic observation (Gram stain, 100×) of the bacterial mixture; Untreated control growth showing dense growth of Gram-negative bacilli (red/pink) **(C)**; PEONE-treated group showing a clear reduction in bacterial density, indicating bactericidal activity and successful resistance modulation **(D)**.

The PEONE was tested against a mixture of *P. aeruginosa* and *K. pneumoniae* using four diluted concentrations. After 24 h, the MIC was determined to be 0.1% v/v (Figure 1A). The MBC was determined to be 0.14% v/v, with a particle size of 1.277 nm, against the tested pathogens (Figure 1B). Gram staining microscopy also confirmed the observed inhibition of bacterial growth (Figures 1C,D).

### 3.3 DNA leakage and protein damage assessed by UV-spectrophotometry

**Figure 2:**
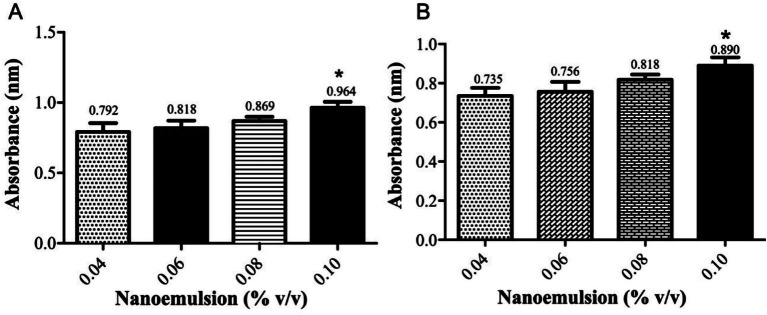
UV–Vis spectrophotometric analysis of bacterial mixture (*K. pneumoniae* and *P. aeruginosa*). **(A)** DNA leakage; **(B)** protein denaturation. The “Control” group refers to the untreated bacterial growth control. *means significant at 0.05.

The study demonstrated that the antibacterial activity of PEONE induced concentration-dependent DNA leakage and protein release into the supernatant from a bacterial mixture of *K. pneumoniae* and *P. aeruginosa*. At a 0.04% concentration, DNA leakage showed an optical density (OD) of 0.792 ± 0.035, while protein denaturation exhibited an OD of 0.735 ± 0.064 (Figure 2). As the NE concentration increased, the levels of leaked DNA and proteins also rose. Notably, at 0.10% concentration, the leakage was significantly higher (*p* < 0.05) compared to other tested concentrations.

### 3.4 Time-kill dynamics

**Figure 3:**
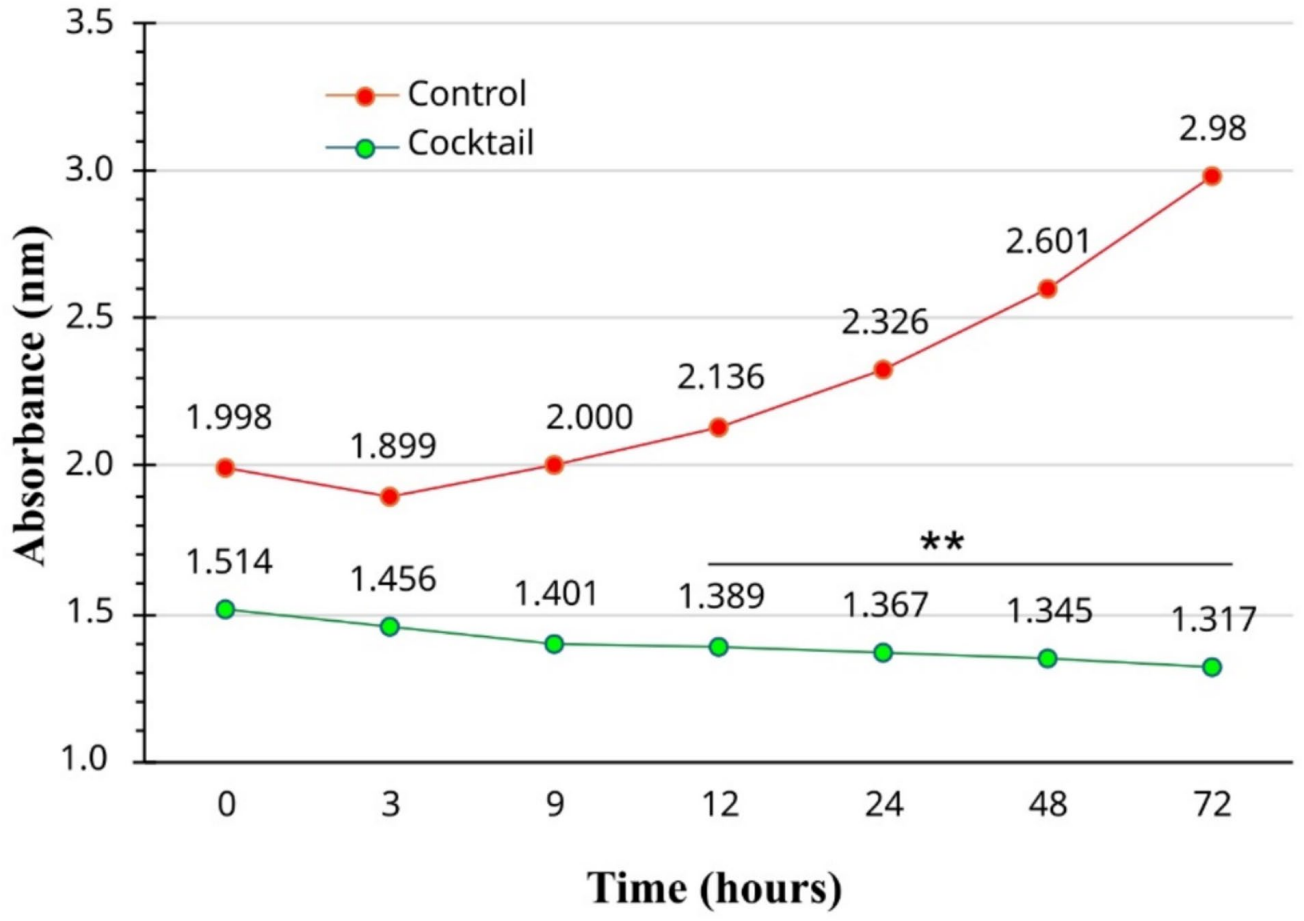
Time-dependent antibacterial activity of 0.1% v/v peppermint essential oil Nano emulsion. The “Control” group refers to the untreated bacterial growth control. **means significant at 0.01.

The antibacterial effect of PEONE increased over time, with a significant decrease in bacterial growth (measured by absorbance) observed between 24 and 72 h (Figure 3). During the first 8 h of incubation, there was no significant difference in optical density (OD) between the control group (without PEONE; OD = 2.00) and the PEONE-treated group (OD = 1.401). However, at 12 h, the control group showed an OD of 2.136, while the PEONE-treated group had an OD of 1.389, representing a significant difference (*p* < 0.05). This trend of significant growth reduction continued consistently until the end of the experiment (72 h) (Figure 3).

### 3.5 Docking results

#### 3.5.1 Identification of Caryophyllene as a high-affinity ligand

The docking results, ranked by predicted binding affinity (ΔG, kcal/mol), are presented in Table 1. Caryophyllene (CID: 5281515) exhibited the strongest binding affinity for both targets, with scores of −9.2 kcal/mol for 4EXY and −7.1 kcal/mol for 6R73, outperforming all other screened compounds. This superior binding energy suggests a high probability of stable and specific interactions within the enzymes’ active sites, identifying it as the most promising candidate for further investigation (Table 2).

**Table 2:** Binding affinities of peppermint oil compounds against β-lactamase targets 4EXY and 6R73.

Complex	Binding affinity (Kcal/mol)	Complex	Binding affinity (Kcal/mol)
4EXY_5281515	−9.2	6R73_5281515	−7.1
4EXY_6432469	−7.9	6R73_27867	−6.2
4EXY_2758	−7.4	6R73_1254	−6.1
4EXY_1254	−7.3	6R73_19243	−6.1
4EXY_26447	−7.2	6R73_2758	−6.1
4EXY_27867	−7.2	6R73_6432469	−6.1
4EXY_19243	−7.1	6R73_7439	−6
4EXY_22311	−6.8	6R73_22311	−5.9
4EXY_7439	−5.8	6R73_26447	−5.8
4EXY_31253	−4.7	6R73_31253	−4.7

#### 3.5.2 Analysis of binding poses and molecular interactions

**Figure 4:**
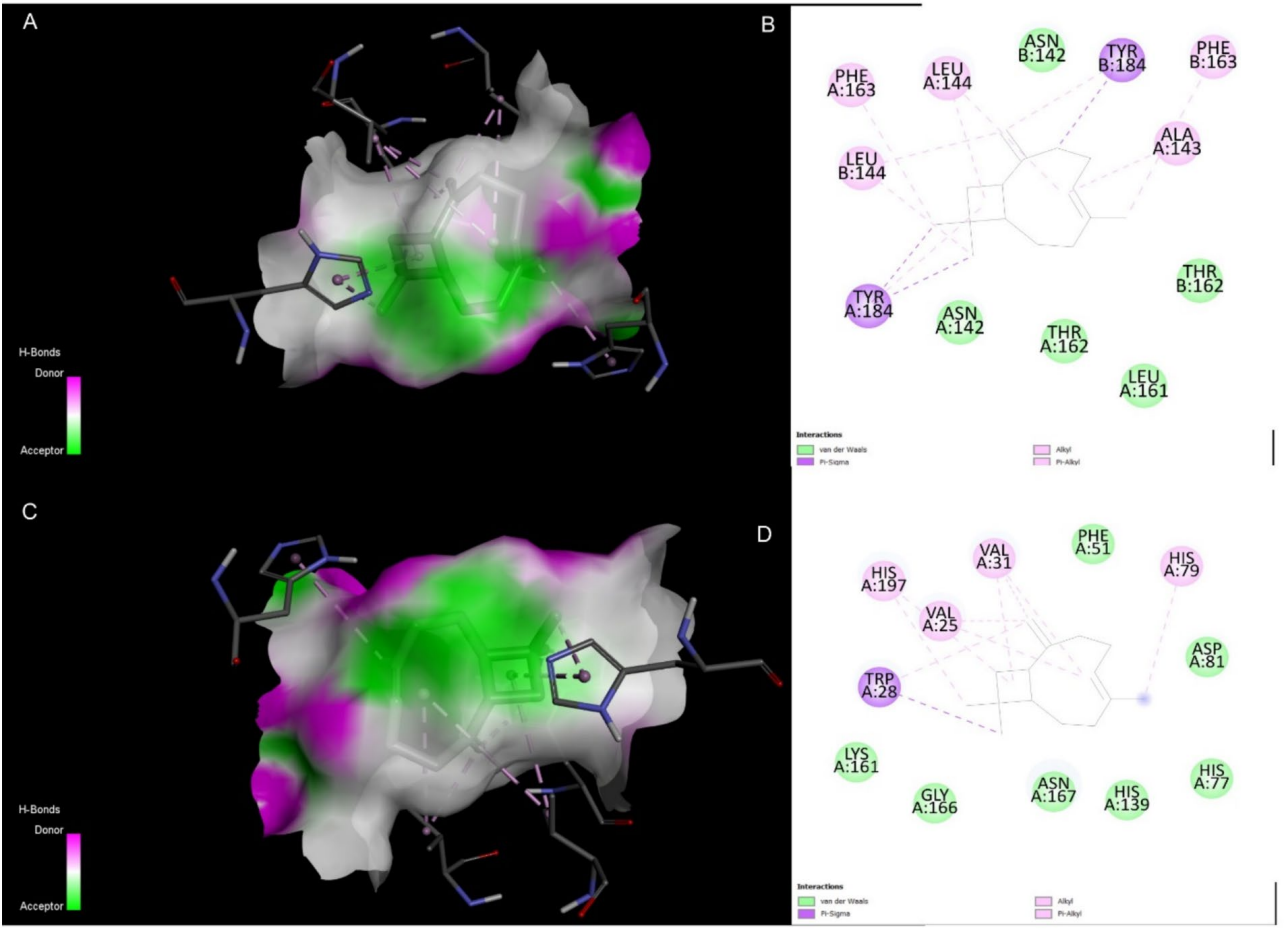
Molecular interactions of Caryophyllene (CID: 5281515) with target β-lactamases. **(A)** 3D representation of the 4EXY_5281515 complex. **(B)** 2D ligand interaction diagram for the 4EXY_5281515 complex. **(C)** 3D representation of the 6R73_5281515 complex. **(D)** 2D ligand interaction diagram for the 6R73_5281515 complex.

The top-ranked docking poses for the Caryophyllene complexes with 4EXY and 6R73 were analyzed to elucidate the molecular basis for the high binding affinity. Visualization of the 3D binding modes revealed that Caryophyllene docks deeply within the active site pocket of both β-lactamases (Figures 4A,C). A non-covalent interactions identified specific contacts stabilizing the complexes (Figures 4B,D). For both targets, the binding was characterized by a high degree of hydrophobic complementarity, consistent with the sesquiterpene structure of Caryophyllene. While specific hydrogen bonding patterns differed between the two proteins, the presence of these key interactions, alongside van der Waals forces and potential pi-pi stacking, corroborates the strong computed binding affinities and suggests a mechanism of inhibition through active site occlusion.

#### 3.5.3 Molecular dynamics simulations confirm complex stability

**Figure 5:**
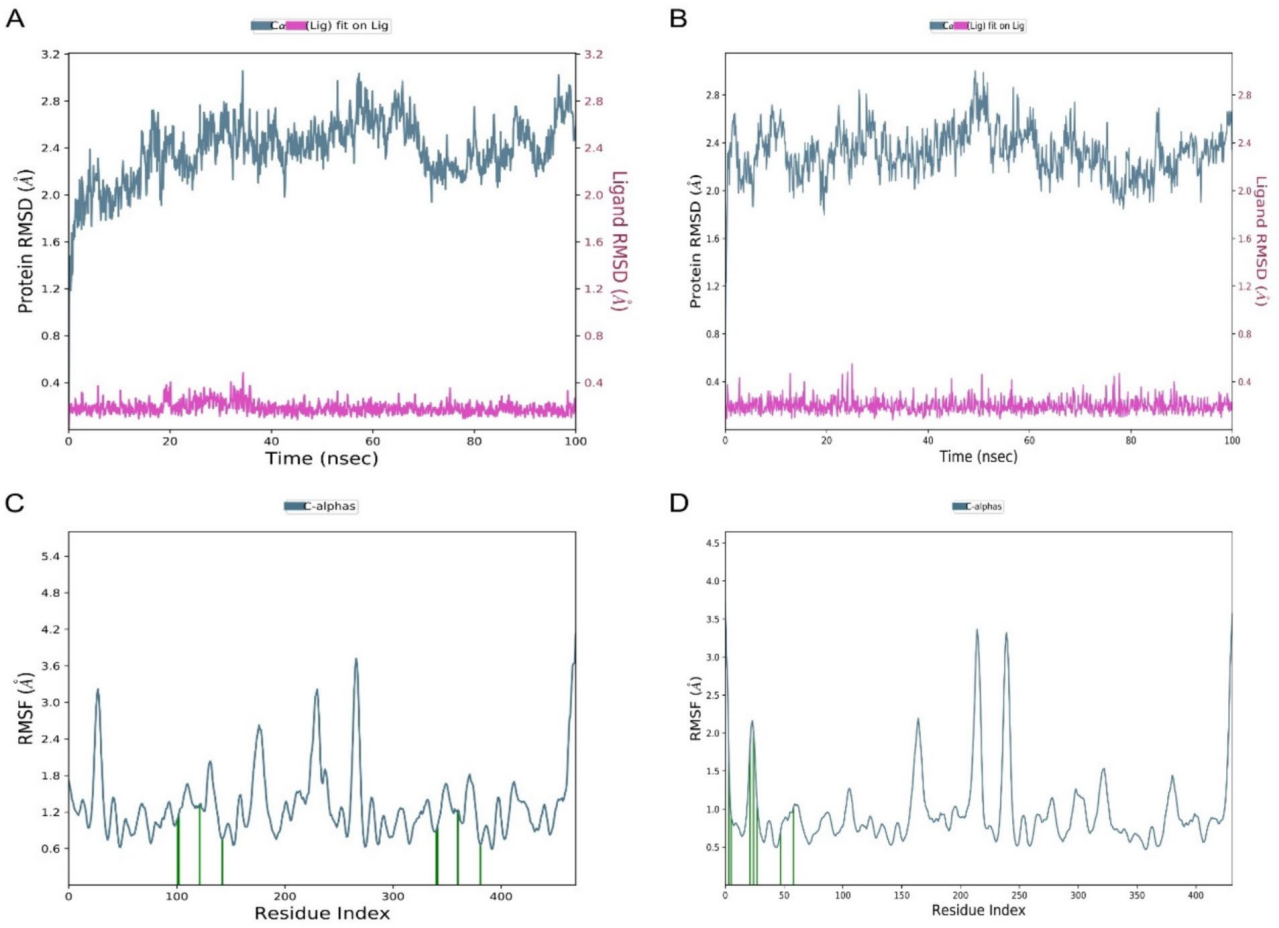
Comparative stability and flexibility analysis from molecular dynamics simulations. **(A,B)** Time-dependent Root Mean Square Deviation (RMSD) of protein alpha carbons (left axis) and ligand atoms (right axis) for complexes **(A)** 4EXY_5281515 and **(B)** 6R73_5281515. **(C,D)** Root Mean Square Fluctuation (RMSF) per residue for the protein in complex with the ligand for systems **(C)** 4EXY_5281515 and **(D)** 6R73_5281515. The residue number is plotted on the X-axis.

To assess the stability and dynamic behavior of the docked complexes, 100 ns molecular dynamics (MD) simulations were performed on both the 4EXY_5281515 and 6R73_5281515 systems. Structural stability was quantified by calculating the RMSD of the protein backbone and the ligand relative to their initial coordinates. For the 4EXY_5281515 complex, the protein RMSD underwent an initial increase during the first ~10 ns, stabilizing thereafter between 2.6–3.0 Å for the remainder of the trajectory (Figure 5A). This suggests a moderate conformational adjustment before achieving a stable equilibrium. Crucially, the ligand RMSD remained consistently low (< 0.4 Å), indicating that Caryophyllene maintained a stable position within the binding pocket despite the protein’s relaxation. The 6R73_5281515 complex exhibited superior global stability, with the protein backbone RMSD fluctuating within a narrower range of 2.0–2.4 Å after equilibration (Figure 5B). The ligand RMSD again remained minimal (< 0.4 Å), confirming a rigid and stable binding mode. The lower overall protein fluctuation in the 6R73 complex suggests a tighter and potentially more rigid binding interaction compared to 4EXY. In both complexes, residues constituting the ligand-binding site exhibited remarkably low fluctuations (< 1.5 Å), demonstrating that Caryophyllene binding imposes a stabilizing effect on the active site architecture. The 4EXY_5281515 complex showed higher flexibility in loop regions and terminal domains (peaks > 3.5 Å), which is typical for solvent-exposed, unstructured regions and does not compromise active site integrity (Figure 5C). The 6R73_5281515 complex displayed a more rigid profile overall, with most residues fluctuating below 2.0 Å (Figure 5D). The reduced magnitude of fluctuations in non-active site regions further supports the conclusion that the 6R73 complex is structurally more stable.

**Figure 6:**
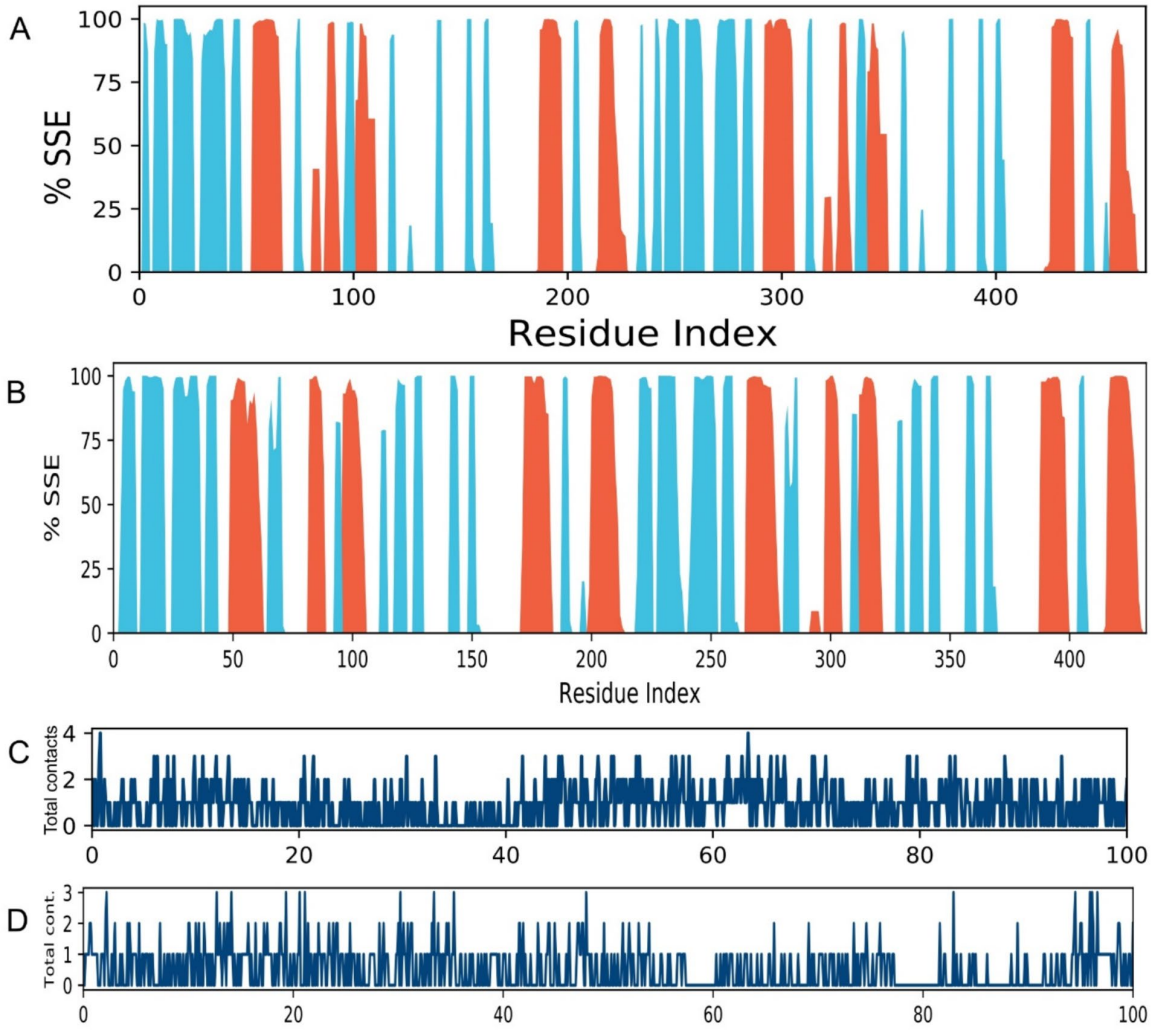
Secondary structure conservation and ligand interaction fingerprints. **(A,B)** Per-residue secondary structure assignment over time for complexes **(A)** 4EXY_5281515 and **(B)** 6R73_5281515 throughout the 100 ns simulation. α-helices and β-sheets are colored red and blue, respectively. **(C,D)** Timeline of specific contacts (e.g., hydrogen bonds, hydrophobic, ionic) between the ligand and protein residue side chains for complexes **(C)** 4EXY_5281515 and **(D)** 6R73_5281515.

The secondary structure elements (SSE) of both proteins remained largely conserved throughout the simulation (Figure 6), indicating that ligand binding did not induce destabilizing unfolding. The preservation of α-helices and β-sheets confirms the stability of the overall protein fold in complex with Caryophyllene. The protein-ligand contacts timeline illustrates a consistent and diverse set of interactions maintained throughout the 100 ns simulation for both complexes (Figures 6C,D). The persistent number of contacts, including hydrogen bonds and hydrophobic interactions, provides dynamic validation of the strong binding observed in the initial docking studies and underscores the stability of the ligand-protein association.

## 4 Discussion

Urinary tract infections (UTIs) represent a major global health burden, with rising antimicrobial resistance (AMR) complicating treatment strategies (Zeng et al., 2022). Among the leading causative agents, *K. pneumoniae* and *P. aeruginosa* are particularly concerning due to their increasing resistance to frontline antibiotics, including β-lactams, fluoroquinolones, and cephalosporins (Bayyiğit et al., 2023; Elfadadny et al., 2024). The failure to accurately identify and target these pathogens exacerbates treatment delays, promotes complications, and accelerates the emergence of resistant strains (Elfadadny et al., 2024). Given the limitations of conventional antibiotics coupled with their adverse effects and growing inefficacy there is an urgent need for alternative antimicrobial agents (Ghosh et al., 2019).

In our study, we explored the efficacy of PEONE as a novel, nanotechnology-driven intervention against MDR *K. pneumoniae* and *P. aeruginosa*. Our findings demonstrate that PEONE exhibits potent antibacterial activity, with a MIC of 0.1% v/v and with an MBC of 0.14% v/v. These results are particularly significant given that the bacterial isolates in our study exhibited resistance to commonly prescribed antibiotics, including Levofloxacin, Penicillin G, Ceftazidime, and Augmentin, aligning with global reports on escalating MDR trends in uropathogens (Gao et al., 2025; de Wet et al., 2025).

Our analyses revealed concentration-dependent DNA leakage and protein denaturation, suggesting that PEONE disrupts bacterial membrane integrity. This mechanism is consistent with prior studies on essential oil nanoemulsions, where small droplet size (~1.277 nm in our formulation) enhances interaction with bacterial cell walls, leading to lysis and leakage of intracellular components (Manzoor et al., 2023). The progressive increase in nucleic acid and protein release at higher PEONE concentrations supports a membrane-disruptive mode of action, which is less likely to induce resistance compared to target-specific antibiotics (Liu et al., 2021). While the antibacterial action of essential oils like peppermint oil is often attributed to non-specific membrane disruption (Lupia et al., 2024), our computational results propose a more targeted, supplementary mechanism for resistance modulation. Molecular docking analysis identified caryophyllene as the constituent exhibiting the strongest binding affinity for the beta-lactamase enzymes 4EXY (*K. pneumoniae*) and 6R73 (*P. aeruginosa*), with scores of −9.2 kcal/mol and −7.1 kcal/mol, respectively. The stability of these ligand-enzyme complexes, validated by molecular dynamics simulations demonstrating low RMSD and RMSF values at the active site, indicates that caryophyllene functions as a potent beta-lactamase inhibitor (Mani et al., 2021). Given that beta-lactamase production is a principal resistance mechanism against penicillin and cephalosporins, the inhibition of these enzymes could potentially resensitize resistant pathogens to conventional antibiotics a cornerstone of β-lactam/β-lactamase inhibitor combination therapy (Almeida-Bezerra et al., 2025). Consequently, the high potency of PEONE against MDR isolates likely stems not only from direct physical membrane damage but also from a synergistic biochemical disruption of critical enzymatic resistance pathways.

Furthermore, our time-kill assay demonstrated a sustained reduction in bacterial viability over 72 h, with a notable decline after an initial 8-h lag phase. This delay may reflect the time required for PEONE to penetrate bacterial biofilms a common virulence factor in chronic UTIs (Raj et al., 2022). The prolonged antibacterial effect suggests that PEONE could be particularly useful in preventing bacterial regrowth, a critical factor in recurrent UTIs.

The rise of MDR underscores the need for innovative therapies (Abdalla et al., 2025). PEONE presents as a promising natural alternative with a multi-target mechanism that may reduce resistance risk, enhanced bioavailability via nanoformulation, and potential for synergistic combinations with conventional antibiotics, potentially lowering required doses and side effects (Mohapatra et al., 2021; Coates et al., 2020).

While antibacterial and docking analyses were conducted on a combined bacterial culture, subsequent research should employ monocultures to elucidate potential species-specific effects. Furthermore, the proposed inhibitory action of caryophyllene on beta-lactamase, strongly supported by *in silico* docking data, necessitates empirical validation through *in vitro* enzymatic assays such as nitrocefin hydrolysis to substantiate the computational findings.

This study demonstrates that PEONE is a potent agent against multidrug-resistant *K. pneumoniae* and *P. aeruginosa*, achieving bactericidal effects through membrane disruption. Crucially, molecular docking and dynamics simulations provide a mechanistic rationale, identifying caryophyllene as a high-affinity inhibitor of beta-lactamase enzymes. However, while our computational results propose a targeted, supplementary mechanism for resistance modulation through beta-lactamase inhibition, it is important to note a key limitation of this study. The proposed inhibitory action of caryophyllene on beta-lactamase, while strongly supported by *in silico* docking and dynamics data, has not been empirically validated through *in vitro* enzymatic assays. Therefore, these findings should be interpreted as a compelling hypothesis generating a mechanistic model for future testing. This dual mechanism of action physical membrane damage and enzymatic resistance modulation makes PEONE a highly promising candidate for combating antibiotic-resistant UTIs. These compelling *in vitro* and *in silico* results strongly justify further investigation, including future work that will be essential to directly confirm beta-lactamase inhibition using assays such as nitrocefin hydrolysis *in vivo* efficacy studies and the development of synergistic formulations with conventional antibiotics. However, while our *in vitro* results are encouraging, further research is necessary to evaluate PEONE’s safety, pharmacokinetics, and efficacy in clinical settings. Specifically, *in vivo* studies assessing urinary mucosal tolerance and long-term antimicrobial effects will be crucial for determining its translational potential in combating MDR uropathogens.

## Data Availability

The original contributions presented in the study are included in the article/Supplementary material, further inquiries can be directed to the corresponding authors.

## Footnotes

1
https://autodock.scripps.edu
