# Supplementary material for: Evaluation of resistance modulation in MDR Pseudomonas aeruginosa and Klebsiella pneumoniae using peppermint oil nanoemulsion: integrating antibacterial assays and molecular modeling
Source: Front Microbiol. 2025 Nov 26;16:1704938. doi: 10.3389/fmicb.2025.1704938 (PMC12689924; doi:10.3389/fmicb.2025.1704938)
Supplement: Supplementary file 1 [file Table_1.docx]

**Table S1**: Chemical composition of the Peppermint essential oil used in this experiment

| **Component** | **Percentage (%)** |
| --- | --- |
| Menthol | 40.7 |
| Menthone | 23.4 |
| 1,8-Cineole | 5.3 |
| (+/-)-Menthyl acetate | 4.2 |
| Isomenthone | 3.7 |
| Menthofurane | 3.7 |
| Neomenthol | 3.2 |
| Limonene | 2.6 |
| Pulegone | 1.9 |
| β-Caryophyllene | 1.7 |
| β-Pinene | 1.1 |
| Germacrene D | 0.9 |
| α-Pinene | 0.7 |
| Piperitone | 0.6 |
| Sabinene | 0.5 |
| neo-iso-Menthol | 0.5 |
| α-Terpineol | 0.4 |
| γ-Terpinene | 0.4 |
| cis-Sabinene hydrate | 0.3 |
| Linalool | 0.3 |
| neo-Menthyl acetate | 0.3 |
| Myrcene | 0.2 |
| 3-Octanol | 0.2 |
| Terpinolene | 0.2 |
| Pulegol | 0.2 |
| Carvone | 0.2 |
| β-Bourbonene | 0.2 |
| (E)-β-Farnesene | 0.2 |
| iso-Menthyl acetate | 0.2 |
| trans-Sabinene hydrate | 0.1 |
| (E)-Anethole | 0.1 |
| β-Elemene | 0.1 |
| α-Thujene | trace (tr) |
| Camphene | trace (tr) |
| α-Phellandrene | trace (tr) |
| (E)-β-Ocimene | trace (tr) |
| iso-Pulegol | trace (tr) |
| iso-Pulegone | trace (tr) |
| Total | 98.3 |
