# Supplementary material for: Evaluation of resistance modulation in MDR Pseudomonas aeruginosa and Klebsiella pneumoniae using peppermint oil nanoemulsion: integrating antibacterial assays and molecular modeling
Source: Front Microbiol. 2025 Nov 26;16:1704938. doi: 10.3389/fmicb.2025.1704938 (PMC12689924; doi:10.3389/fmicb.2025.1704938)
Supplement: Supplementary file 2 [file Supplementary_file_1.docx]

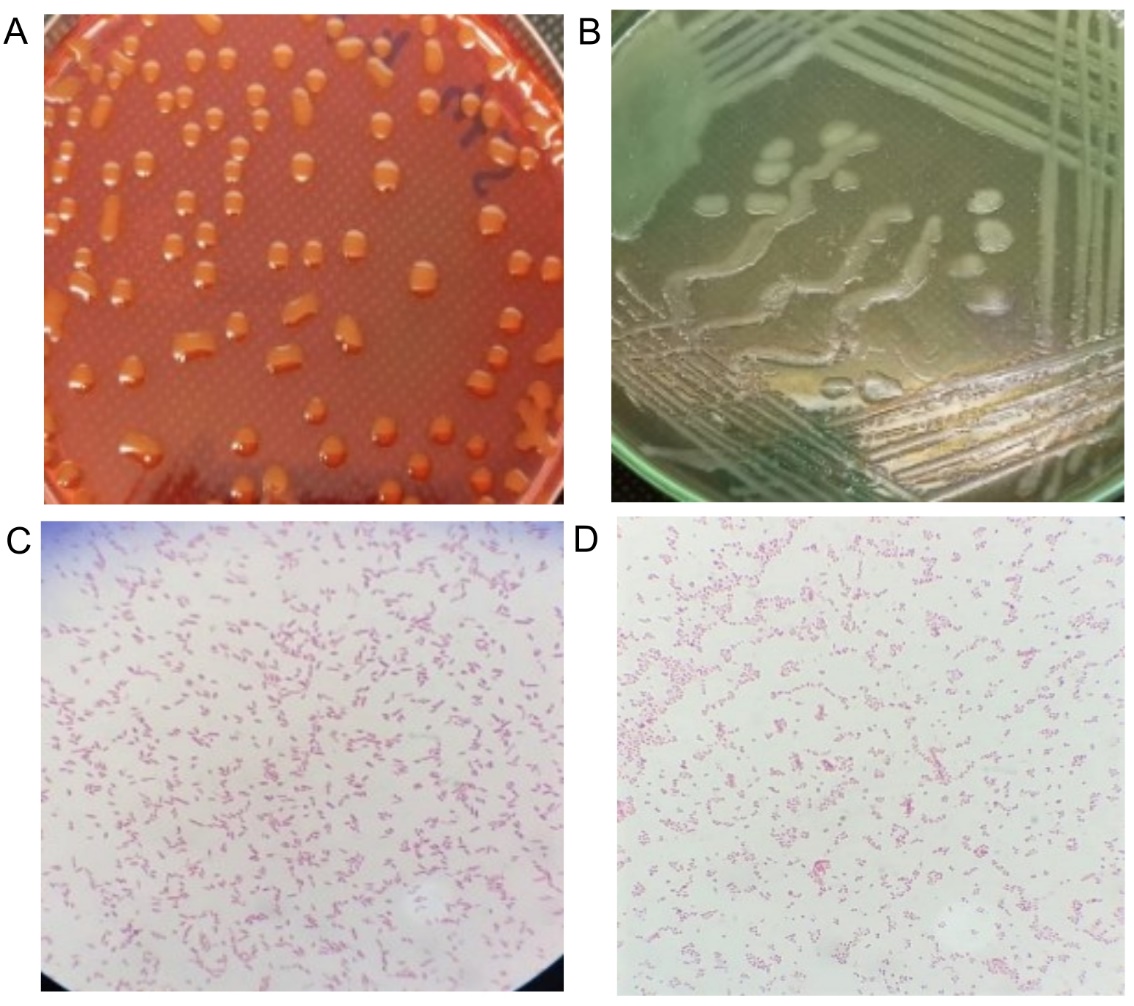


**Figure S1:** Colonies of *P. aeruginosa* on Mueller-Hinton agar (A) and *K. pneumoniae* on Cystine Lactose Electrolyte-Deficient agar (B). Gram-stained microscopic images of *P. aeruginosa* (C) and *K. pneumoniae* (D).
